# Recent advances in transdermal drug delivery systems: a review

**DOI:** 10.1186/s40824-021-00226-6

**Published:** 2021-07-28

**Authors:** Woo Yeup Jeong, Mina Kwon, Hye Eun Choi, Ki Su Kim

**Affiliations:** School of Chemical Engineering, Pusan National University, 2 Busandaehak-ro 63 beon-gil, Geumjeong-gu, Busan, 46241 Republic of Korea

**Keywords:** Transdermal drug delivery, Skin, Active/passive method, Characterization

## Abstract

Various non-invasive administrations have recently emerged as an alternative to conventional needle injections. A transdermal drug delivery system (TDDS) represents the most attractive method among these because of its low rejection rate, excellent ease of administration, and superb convenience and persistence among patients. TDDS could be applicable in not only pharmaceuticals but also in the skin care industry, including cosmetics. Because this method mainly involves local administration, it can prevent local buildup in drug concentration and nonspecific delivery to tissues not targeted by the drug. However, the physicochemical properties of the skin translate to multiple obstacles and restrictions in transdermal delivery, with numerous investigations conducted to overcome these bottlenecks. In this review, we describe the different types of available TDDS methods, along with a critical discussion of the specific advantages and disadvantages, characterization methods, and potential of each method. Progress in research on these alternative methods has established the high efficiency inherent to TDDS, which is expected to find applications in a wide range of fields.

## Introduction

Drug delivery system (DDS) is a generic term for a series of physicochemical technologies that can control delivery and release of pharmacologically active substances into cells, tissues and organs, such that these active substances could exert optimal effects [1, 2]. In other words, DDS covers the routes of administration and drug formulations that efficiently deliver the drug to maximize therapeutic efficacy while minimizing any side effect [3–5]. Depending on the delivery route, there are many types of administration modalities, such as oral administration, transdermal administration, lung inhalation, mucosal administration, and intravenous injection. Among them, the transdermal drug delivery system (TDDS) represents an attractive approach.

TDDS has become one of the most widely investigated routes of noninvasive drug delivery into the body through the skin, unlike conventionally used direct administration routes that make use of needle-based injections. TDDS has significantly influenced the delivery of various therapeutic agents, especially in pain management, hormonal therapy, and treatment of diseases of the cardiovascular and central nervous systems [6–9]. TDDS does not involve passage through the gastrointestinal tract; therefore, there is no loss due to first-pass metabolism, and drugs can be delivered without interference from pH, enzymes, and intestinal bacteria. In addition, TDDS can be used to control drug release according to usage restrictions, thereby contributing to the high persistence of this method. Most importantly, because TDDS is a noninvasive administration method and involves minimal pain and burden on the patient, drugs can be safely and conveniently administered to children or the elderly [10–12].

**Fig. 1:**
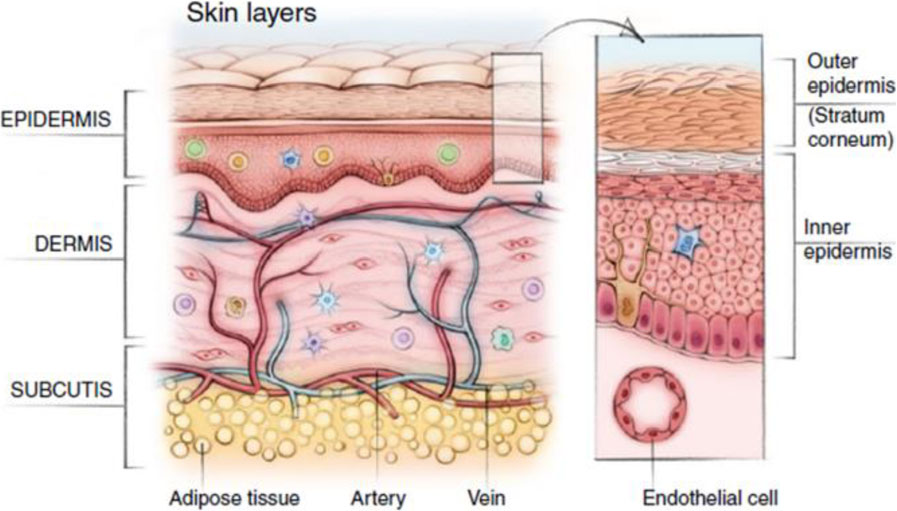
The structure of skin

However, it still does not utilize its full potential due to the innate skin barrier. The skin is the outermost organ with a multi-layered structure, and the role of the skin is to protect our body by blocking environmental hazards such as chemicals, heat, and toxins [13, 14]. (Fig. 1). Such skin can be divided into the epidermis, which has the protective function, and the dermis, where blood vessels are located, and produces skin cells, and each layer has elements that interfere with transdermal delivery.

First, the skin barrier effect of the epidermis occurs in the stratum corneum, the outermost layer, and is a property of blocking external substances. The barrier effect is very significant in the transport of substances having a large molecular weight. In TDDS, it is generally accepted that the delivery of substances with small molecular weights utilizes the intracellular pathway. However, for substances having a large molecular weight, methods and various mechanisms using the intracellular pathway in addition to the intercellular pathway are introduced and used [15–17]. This is due to the structure of the skin because the part called lipid containing both cells and hydrophilic substances and hydrophobic substances does not have a perfectly regular position but exists with regularity [18]. These structural features can be explained by the principles of physicochemical properties that are attempted to enhance drug delivery through the skin. Next, the vascular system in the dermal layer can inhibit transdermal delivery. A one-cell-thick layer of endothelial cells terminating in the papillary loops of the superficial arteriovenous plexus near the dermal-epidermal junction in the upper dermis represents the interface between the tissues surrounding the skin and the human vasculature. The role of the endothelium in the skin is like that of the whole body. It actively responds to pressure, shear, osmotic pressure, heat, chemokines, and cytokines by modulating permeability and inducing vasodilation or constriction [19]. Therefore, the biggest issue of TDDS is to resolve the barrier effect of the stratum corneum, deliver the drug to the skin tissue, and pass through the cellular and vascular tissue to reach the target tissue. The problem is that only a small amount of the drug can be delivered through the skin tissue [20, 21].

To solve this problem, various novel TDDS techniques have been intensively developed and have emerged as attractive administration methods. In addition, such development could represent a competitive advantage over other drug administration methods in terms of the delivered dose, cost-effectiveness, and therapeutic efficacy [21–24].

Here, we review various transdermal drug delivery techniques (Table 1). We summarize the characteristics of active/passive transdermal delivery and characterization methods. In addition, we discuss future perspectives in the field of TDDS.

**Table 1:** The advantages and disadvantages of various transdermal delivery system

	Method	Advantages	Disadvantages	Ref.
**Active Delivery**	**Iontophoresis**	• Improving the delivery of polar molecules as well as high molecular weight compounds • Faster and easier administration • Enabling continuous or pulsatile delivery of drug	• Risk of burns if electrodes are used improperly • Difficulty stabilizing the therapeutic agent in the vehicle • Complexity of the drug release system	[25–29]
	**Sonophoresis**	• Allows strict control of transdermal diffusion rates • In many cases, greater patient approval • Less risk of systemic absorption • Helpful to break up blood clots • Not immensely sensitizing	• Can be prolonged to administer • Minor tingling, irritation and burning • SC must be unbroken for effective drug penetration	[30–33]
	**Electroporation**	• Highly effective, reproducible, directed drug transfer • Permits rapid termination of drug delivery through termination • Not immensely sensitizing	• Impossible to use on a large area • Can be disturb the cargo if high voltage is uesd • Possibility of cell damage • Relatively nonspecific	[34–36]
	**Photomechanical waves**	• Can improve transfer of molecules across the plasma membrane of cells in vitro without loss of viability • Not appear to cause injury to the viable skin • Do not cause pain or discomfort	• Lack of human clinical data	[37–40]
	**Microneedle**	• Painless administration of the active pharmaceutical ingredient • Faster healing at injection site • No fear of needle • Specific skin area can be targeted for proper drug delivery	• Lower dosing accuracy than hypodermic needles • Penetration depth of various particles depending on the skin layer • Possibility of venous collapse due to repeated injections	[41–54]
	**Thermal ablation**	• Avoid the pain, bleeding, and infection • Can remove SC selectively without damaging deeper tissues • Better control and reproducibility • Low cost and disposable device	• Structural changes in the skin must be evaluated • Existing concerns about the use of extreme temperatures and the logistics of such devices	[55–59]
**Passive delivery**	**Vesicles**	• Accomplish sustained drug release behavior • Control the absorption rate through a multilayered structure	• Chemically unstable • Expensive of formulations • Limitation of drug loading	[60–63]
	**Polymeric nanoparticles**	• Accomplish targeted and controlled release behavior • High mechanical strength and non-deformability • Can be made of various biodegradable materials • Can be loaded both hydrophilic and hydrophobic drugs • Can avoid the immune system due to small size	• Difficult to break down • Not enough toxicological assessment has been done • Some processes are difficult to scale up	[64-70]
	**Nanoemulsion**	• Long-term thermodynamic stability • Excellent wettability • High solubilization capacity and physical stability • Possible to formulate it in variety of formulations	• Requires large concentration of emulsifiers • Limited solubilizing capacity for high-melting substances • Variable kinetics of distribution processes and clearance	[58, 71, 72]

## Enhancement of transdermal delivery by equipment (active delivery)

**Fig. 2:**
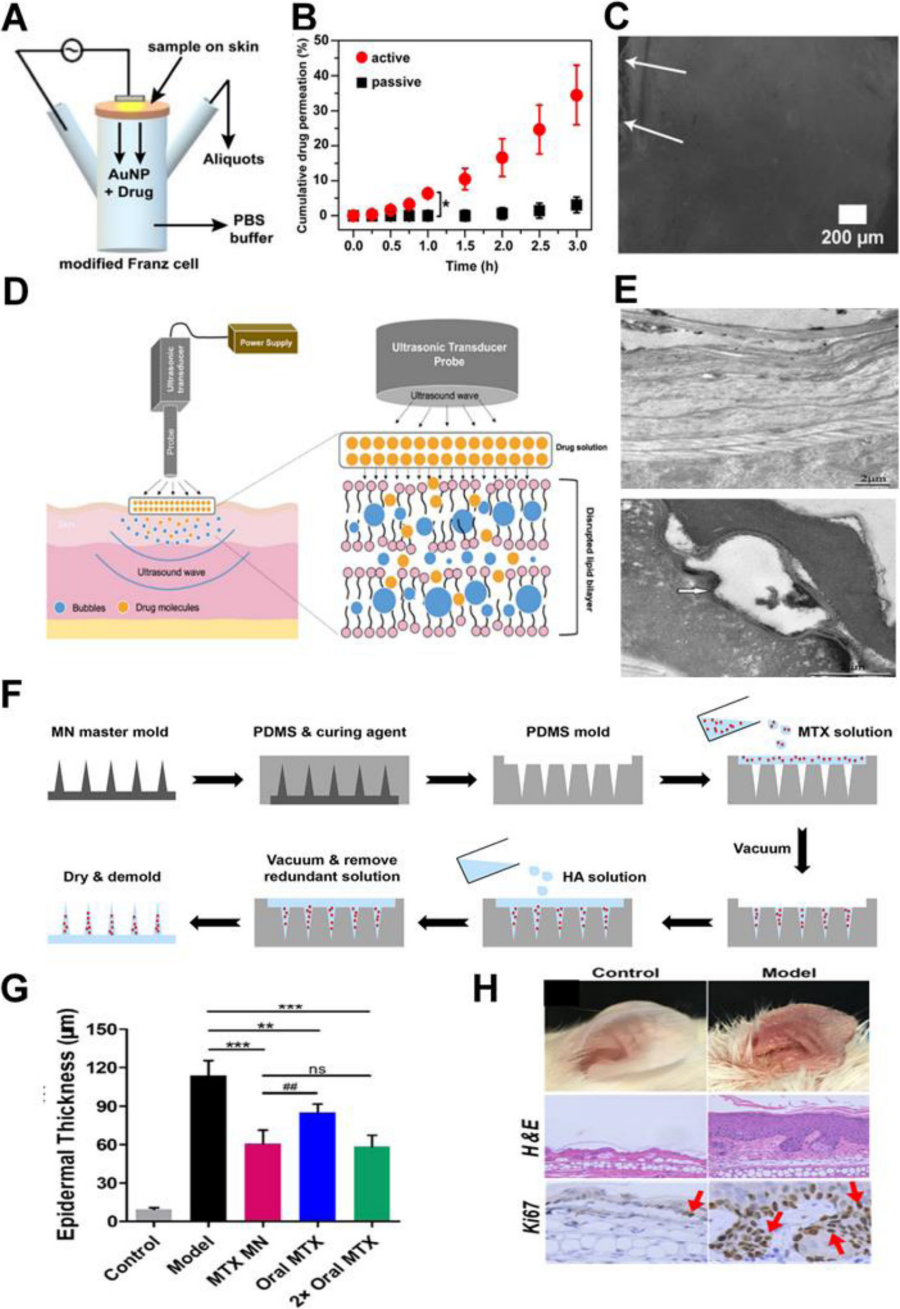
**A** Experimental set up for skin permeation test using iontophoresis. **B** In vitro drug release profiles of drug-loaded AuNP oleogels (d-AuNP) on skin. **C** Fluorescence spectroscopy images obtained from skin permeation experiment after 1 h of application. Arrows mark the top surface of the skin segment treated with d-AuNP. **D** A schematic illustration of sonophoresis-assisted transdermal drug delivery. **E** Penetration pathways of LaNO3 after treatment with low frequency sonophoresis, and TEM images of SC after treatment with low frequency sonophoresis using RuO4 fixation in the absence of low frequency sonophoresis (left) and after 5 min (middle) and 10 min (right) of treatment with low frequency sonophoresis. **F** Schematic Illustration Showing the Fabrication Process of the MTX-Loaded HA-Based Dissolving MN Patch. **G** Quantitative analysis of epidermal thickness. **H** Therapeutic effects of MTX-loaded MNs and oral administration of the same dose and a double dose of MTX on IMQ-induced psoriasis-like skin inflammation. Representative photographs of left ear lesions and skin sections stained with H&E and Ki67 on day 7. **A**, **B**, **C** Reproduced from [29], copyright permission by American Chemical Society 2020. **D** Reproduced from [74], copyright permission by Springer Nature 2021. **E** Reproduced from [40], copyright permission by Elsevier 2010. **F**, **G**, **H** Reproduced from [54], copyright permission by American Chemical Society 2019

External stimuli, such as electrical, mechanical, or physical stimuli, are known to enhance skin permeability of drugs and biomolecules, as compared to the delivery of drugs by topical application on the skin [73]. TDDS supplemented by appropriate equipment is termed as active transdermal delivery, which is known to deliver drugs quickly and reliably into the skin. In addition, this mode of enhanced TDDS can accelerate the therapeutic efficacy of delivered drugs (Fig. 2) [75–77].

### Iontophoresis

Iontophoresis promotes the movement of ions across the membrane under the influence of a small externally applied potential difference (less than 0.5 mA/cm^2^), which has been proven to enhance skin penetration and increase release rate of several drugs with poor absorption/permeation profiles. This technique has been utilized in the in vivo transport of ionic or nonionic drugs by the application of an electrochemical potential gradient [25]. The efficacy of iontophoresis depends on the polarity, valency, and mobility of the drug molecule, the nature of the applied electrical cycle, and the formulation containing the drug. In particular, the dependence on current makes drug absorption through iontophoresis less dependent on biological parameters, unlike most other drug delivery systems (Fig. 2A, B) [26]. This modality could additionally include electronic means of reminding patients to change dosages, if desired, to increase patient compliance [27, 28].

### Sonophoresis

The desired range of ultrasound frequencies generated by an ultrasound device can improve transdermal drug delivery [30, 31]. Low-frequency ultrasound is more effective, because it facilitates drug movement by creating an aqueous path in the perturbed bilayer through cavitation (Fig. 2C) [32]. The drug under consideration is mixed with a specific coupler, such as a gel or a cream, which transmits ultrasonic waves to the skin and disturbs the skin layers, thereby creating an aqueous path through which the drug can be injected. Drugs typically pass through passages created by the application of ultrasonic waves with energy values between 20 kHz and 16 MHz. Ultrasound also increases the local temperature of the skin area and creates a thermal effect, which further promotes drug penetration. Several drugs of different classes have been delivered by this method regardless of their solubility, dissociation and ionization constants, and electrical properties (including hydrophilicity), such as mannitol and high molecular weight (MW) drugs such as insulin. However, the exact mechanism of drug penetration through this method is not yet completely understood, and problems with device availability, optimization of duration of exposure and treatment cycles for delivery, and undesirable side effects including burns persist.

### Electroporation

This method uses the application of high voltage electric pulses ranging from 5 to 500 V for short exposure times (~ms) to the skin, which leads to the formation of small pores in the SC that improve permeability and aid drug diffusion [34, 35]. For safe and painless drug administration, electric pulses are introduced using closely positioned electrodes. This is a very safe and painless procedure involving permeabilization of the skin and has been used to demonstrate the successful delivery of not only low MW drugs, such as doxorubicin, mannitol, or calcein, but also high MW ones such as antiangiogenic peptides, oligonucleotides, and the negatively charged anticoagulant heparin. However, this method has the disadvantages of small delivery loads, massive cellular perturbation sometimes including cell death, heating-induced drug damage, and denaturation of protein and other biomacromolecular therapeutics.

### Photomechanical waves

Photodynamic waves transmitted to the skin can penetrate the SC, allowing the drug to pass through the transiently created channel [37, 39]. The incident wave produces limited ablation, which is achieved by low radiation exposure of approximately 5–7 J/cm^2^ to increase the depth to 50–400 μm for successful transmission. This limited ablation showed a longer increase and duration as compared to that in other direct ablation techniques, which made it necessary to control properties of the photodynamic waves to ensure delivery of the product to the intended depth in the skin. The wave generated by a single laser pulse also showed increased skin permeability within minutes, allowing macromolecules to diffuse into the skin. Dextran macromolecules of 40 kDa weight and 20 nm latex particles could be delivered by a single photodynamic laser pulse of a 23-ns duration.

### Microneedle

The microneedle drug delivery system is a novel drug delivery system, in which drugs are delivered to the circulatory system through a needle [41]. This represents one of the most popular methods for transdermal drug delivery and is an active area of current research. This involves a system in which micron-sized needles pierce the superficial layer of the skin, resulting in drug diffusion across the epidermal layer. Because these microneedles are short and thin, these deliver drugs directly to the blood capillary area for active absorption, which helps in avoiding pain [42]. Scientists have attempted to use multiple techniques for appropriate optimization and geometric measurements required for effective insertion of microneedles into human skin, which also represents the broad objective of research on microneedles.

The fabrication of microneedle system has been widely investigated with considering the objective, drug type and dose, and targets for use [43]. Up to now, the microneedle can be fabricated with laser-mediated techniques and photolithography. The laser-mediated fabrication techniques are used for manufacturing metal or polymer microneedle. The 3D structure of a microneedle is generated through cutting or ablating on a flat metal/polymer surface using a laser [44, 45]. Photolithography is known as the method of elaborately fabricating microneedle and has the advantage of being able to manufacture needles of various shapes using various materials. This method is mainly used to manufacture dissolving/hydrogel microneedles or silicon microneedles via making an inverse mold based on the microneedle structure through etching of photoresist [46]. In addition, 3D printing [47], Microstereolithography [48], and Two-photon polymerization [49] are also investigated for preparing various microneedle system.

The prepared microneedles could be of several types, such as solid microneedles that simply make a physical path through which drugs can be absorbed, drug-coated microneedles which facilitate delivery of drugs coated on the surfaces of the needles as the latter enter the skin, dissolving microneedles made of drug formulations that dissolve in the body, naturally delivered melting needles which involve drug storage in hollow needles followed by administration (such as a specific injection type), and microneedle patches combined with diverse patch types (Fig. 2D, E) [50–54].

### Thermal ablation

Thermal ablation, also known as thermophoresis, is a promising technique for selectively disrupting the stratum corneum structure by localized heat which provides enhanced drug delivery through microchannels created in the skin [55]. To ablate the stratum corneum by thermal ablation, a high temperature above 100 °C is required and this leads to heating and vaporization of keratin. Additionally, the degree of alteration of the stratum corneum structure is proportional to the locally elevated temperature, indicating that it is an ideal technique for precise control of drug delivery. The thermal exposure should be short within microseconds to create a high enough temperature gradient across the skin for selective ablation of the stratum corneum without damaging the viable epidermis. Micron-scale defects created from thermal ablation are small enough (50–100 μm in diameter) to avoid the potential to cause pain, bleeding, irritation, and infection. Therefore, the patient is well tolerated if there is no damage to the cells of the deeper tissues. In addition, thermal ablation has better control and reproducibility than other approaches such as mechanical abrasion, chemical treatment, or tape-stripping. And it offers effective delivery of small molecules as well as high molecular weight compounds. However, the structural changes in the skin must be evaluated, especially when using higher energy for enhancing the diffusion rate of drug molecules.

Thermal ablation can usually be induced by laser and radiofrequency methods depending on the different sources of thermal energy [56, 57]. Laser thermal ablation methodologies utilize a laser to induce micropore structure of skin as well as the increase of the skin temperature which increases skin diffusivity. Laser light energy is absorbed by water and pigments of the skin and transforms to thermal energy leading to water excitation and explosive evaporation from the epidermis. The degree of the ablated skin depth can be precisely controlled upon tuning many parameters such as wavelength, pulse length, energy, number and repetition rate, tissue thickness, absorption coefficient, and duration time of laser exposure. Laser thermal ablation, especially when using Er:YAG laser, makes it possible to increase the penetration of drugs by more than 100 times and enhance the delivery of both lipophilic and hydrophilic drugs including biomacromolecules such as peptides, proteins, vaccines, and DNAs [56–58].

Radiofrequency thermal ablation involves the placement of an array of needle-like metallic microelectrodes directly onto skin and application of high frequency electric current into the skin in radiofrequency range (100–500 kHz) which produce micron-scale pathways in stratum corneum. Exposure of the skin to a high radiofrequency causes ionic vibrations within the tissue leading to the generation of localized heat in specific areas of the skin. And thus, induced heat cause water evaporation and ablates the cells of the epidermis under each filament creating microchannels up to 50 μm in depth. The process is completed within a few seconds and microchannels are filled with intestinal fluid through which hydrophilic molecules can permeate. The rate of drug delivery is proportional to the degree of the ablated skin depth which is controlled by the size and density of the microchannels. Radiofrequency thermal ablation can sustain the drug release and enhance the delivery of a wide range of drugs with hydrophilic nature including macromolecules using a low-cost, disposable device [59].

## TDDS using chemical enhancers (passive delivery)

To achieve enhanced transdermal delivery and therapeutic efficacy, drugs should have low MW (less than 1 kDa), an affinity for lipophilic and hydrophilic phases, short half-life, and a lack of skin irritability [64]. Many factors affect drug penetration through the skin, such as species differences, skin age and site, skin temperature, state of the skin, area of application, duration of exposure, moisture content of the skin, pretreatment methods, and physical characteristics of the penetrant.

**Fig. 3:**
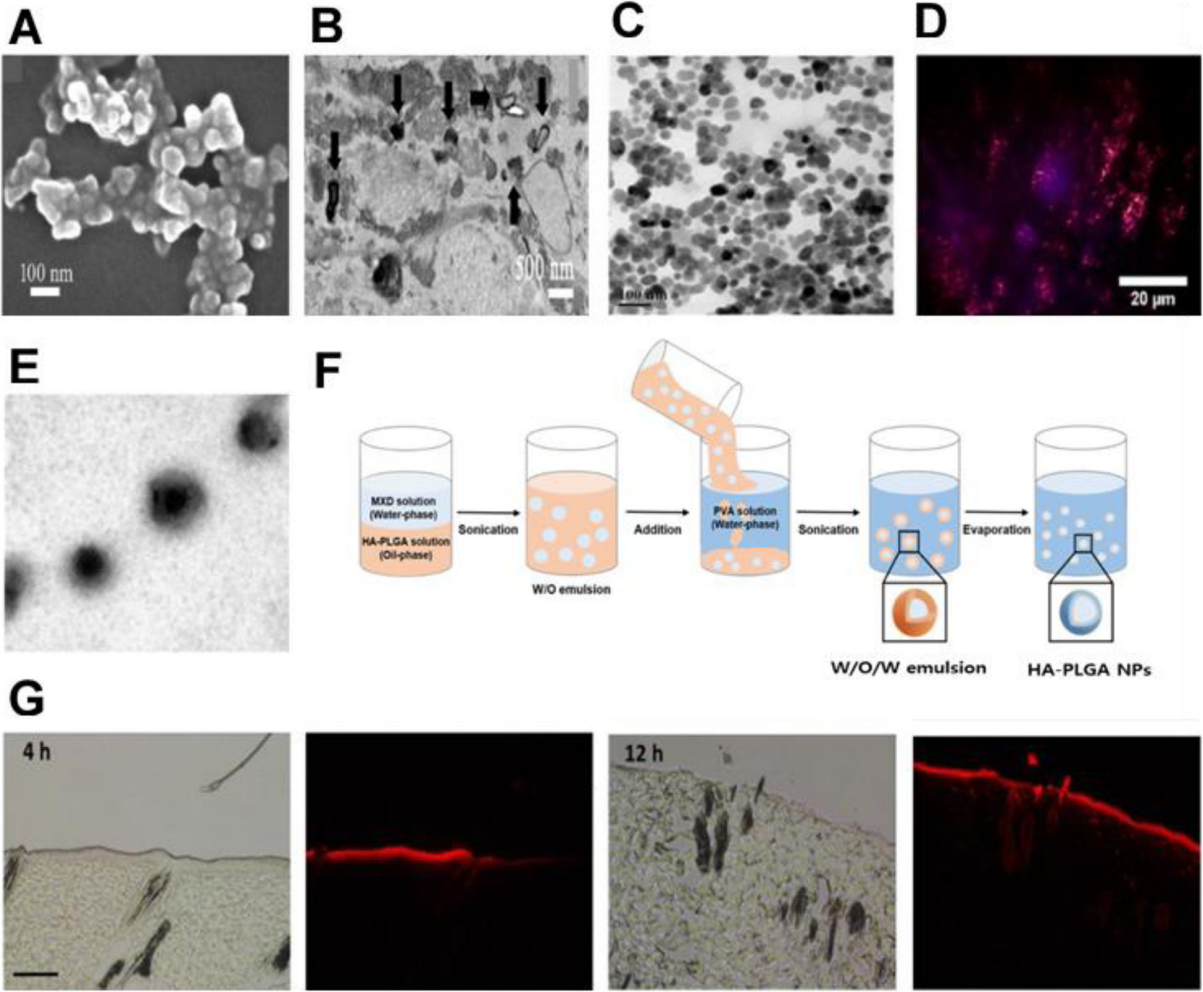
**A** SEM images of the prepared ALA-ES gels. **B** TEM images of ALA-ES in human HS tissue dermis. ALA-ES is indicated using black arrows. **C** TEM image of OA-UCNP. The nanoparticles show near-spherical shape with an average diameter around 25 nm. **D** Microscopy images of a section of a sample pig ear skin under 980 nm excitation laser. **E** Schematic illustration of W/O/W emulsification of HA-PLGA. **F** Fluorescence microscopic images of histological sections of rat skin at 4 and 12 h after topical application of Rho B-encapsulated HA-PLGA NPs. Scale bar, 100 μm. **A**, **B** Reproduced from [63], copyright permission by American Chemical Society 2018. **C**, **D** Reproduced from [67], copyright permission by IOP Publishing Ltd. 2020. **E**, **F**, **G** Reproduced from [68], copyright permission by BioMed Central Ltd. 2019

Recent studies that have focused on aspects of transdermal drug delivery technologies ranging from the development of chemical enhancers that increase the spread of drugs across the skin or increase the solubility of drugs in the skin to novel innovative approaches that extend this concept to the design of super-strong formulations, microemulsions, and vesicles [65, 66] (Fig. 3). Penetration enhancers can be used alone or in combination with chemical penetration enhancers with proven superior skin penetration as compared to that of individual chemicals. These synergistic systems include eutectic mixtures and nanoparticle composite self-assembled vesicles. Therefore, research in recent years have focused on the application of suitable molecular simulation methodologies in understanding the skin lipid barrier, mechanisms regulating penetration of molecules across the skin and transport of penetration enhancers, and perturbations in the skin barrier function.

### Vesicles

Vesicles are colloidal particles filled with water and consist of amphiphilic molecules in a bilayer arrangement. Under conditions of excess water, these amphiphilic molecules form concentric bilayers with one or more shells (multilayer vesicles). Vesicles can carry water-soluble and fat-soluble drugs to achieve transdermal absorption. When utilized for topical applications, vesicles can be used to achieve sustained release of stored drugs. It is also possible to employ vesicles in TDDS to control the absorption rate through a multilayered structure. Owing to the presence of different components, vesicle systems can be divided into several types, such as liposomes, transfersomes, and ethosomes, depending on the properties of the constituent substances [60].

Liposomes are circular soft vesicles formed by one or more bilayer membranes that separate an aqueous medium from another. Their main components are usually phospholipids, with or without cholesterol. Phospholipid molecules are mainly composed of different polar head groups and two hydrophobic hydrocarbon chains. Polar groups can be either positively or negatively charged. Hydrocarbon chain molecules have different lengths and different degrees of unsaturation. The formation of liposomes occurs spontaneously upon reconstitution of a dry lipid film in an aqueous solution. This unique structure allows liposomes to be both hydrophilic and hydrophobic and affords encapsulation of both water-soluble and fat-soluble substances. However, some studies have shown that liposomes can only remain on the surface of the skin and cannot pass through the granular layer of the epidermis, thereby minimizing the amount of drug absorbed into the blood circulation. This property increases the retention of drugs that stay on the skin, prolong their activity at the site of the lesion, and allow long-term sustained release. Therefore, liposomes are the preferred system of choice for the topical treatment of skin diseases [61].

Transfersomes are also called deformable liposomes, or elastic or highly flexible liposomes. The most important feature of these vesicles is the elasticity that results from the addition of single-chain surfactants. These surfactants make the phospholipid bilayer fluid and vesicles highly deformable, thereby rendering these into first-generation transfersomes. Over time, second-generation transfersomes have emerged, consisting of at least one basic bilayer building block (typically fluid-phase phosphatidylcholine lipids) and at least two or more polar lipophilic substances. Third-generation transfersomes are a combination of amphiphilic surfactants, with or without phospholipids. The possibility of deformation has facilitated the design of transfersomes to those capable of penetrating skin pores 5 to 10 times smaller than their size to enable delivery of skin-penetrating drugs with MW up to 1000 kDa. In addition, TDDS using transfersomes allows the administration of macromolecular drugs such as peptides or proteins [62].

Ethosomes are composed of phospholipids, alcohols, and water. Compared with liposomes, ethosomes have higher alcohol concentrations. Ethosomes promote the percutaneous penetration of drugs, with phospholipids also contributing to the process [63] (Fig. 3A, B). The flexibility and fluidity of ethosomes increase as water molecules near the lipid headgroup are replaced by alcohol. Ethosomes have the characteristic size of small particles, a stable structure, and a high capture efficiency that can delay drug release; therefore, compared with regular liposomes, ethosomes can transport drugs with deep penetration or directly through the skin. These formulations are also known to greatly improve drug release into the circulating blood and drug transdermal efficacy. Ethosomes are a type of multiphase dispersion system characterized by better stability and a longer retention period than those of transfersomes.

### Polymeric nanoparticles

Nanoparticles (NPs) are nanocarriers with sizes ranging between 1 and 1000 nm and can be classified into several types according to their composition. Drug administration in the form of NPs leads to targeted and controlled release behavior, changes in in vivo dynamics of the drug, and extends the drug residence time in the blood, which further lead to improved drug bioavailability and reduced toxicity and side effects. NPs are conventionally generated by polymerization and crosslinking, and biodegradable polymeric materials such as gelatin and polylactic acid (PLA) are often used [67, 69, 70]. In the field of TDDS, polymeric NPs are gaining increased attention because they can overcome the limitations of other lipid-based systems, such as by conferring protection to unstable drugs against degradation and denaturation and achieving continuous drug release to reduce side effects. Increase in the concentration gradient improves transdermal penetration of the drug. Depending on the manufacturing method and structure, polymeric NPs can be classified as nanospheres, nanocapsules, and polymer micelles. Widely used polymers include polylactic acid, poly(D,L-lactide-co-glycolide) (PLGA), polycaprolactone, polyacrylic acid, and natural poly esters (including chitosan, gelatin, and alginate). These polymer chains can be synthesized by covalent linkage of two or more single polymeric units under specific conditions, such as the presence of a synthetic membrane that mimics the cellular lipid bilayer membrane. Although these polymers can form a complex structure, the polymer membrane is highly structured owing to the high MW polymer chains; for this reason, polymeric NPs, characterized by high mechanical strength and non-deformability cannot pass through pores with dimensions smaller or equal to their size. However, these NPs can be difficult to break down, which means drugs can be stored for a substantially long period, followed by its release from the NPs and diffusion into deeper layers of the skin (Fig. 3C-G) [68, 78–80].

### Nanoemulsion

Nanoemulsions are a mixture characterized by low viscosity and isotropic, thermodynamic, and dynamic stability [71]. The mixture consists of transparent or translucent oil globules dispersed in an aqueous phase stabilized by an interfacial membrane formed by surfactant or co-surfactant molecules of extremely small droplet size. The particle size of commonly used nanoemulsions ranges from 100 to 1000 nm, although an upper limit to the particle size has been proposed on account of its nanoscale dimensions. Nanoemulsions are different from microemulsions; although nanoemulsions have almost the same droplet size range, composition, and appearance as microemulsions, they differ greatly in terms of structural aspects and long-term thermodynamic stability. The small particle size, large specific surface area, and low surface tension of nanoemulsions provide excellent wettability that ensures close contact with the skin. In addition, nanoemulsions offer many other benefits such as high solubilization capacity and physical stability, improved bioavailability, ease of preparation, production with less energy input, and long shelf life. Nanoemulsions exhibit a shorter transdermal time and better transdermal absorption than commonly used topical skin preparations. Depending on the composition, nanoemulsions can include oil-in-water (O/W: oil phase dispersed in a continuous aqueous phase), water-in-oil (W/O: aqueous phase dispersed in a continuous oil phase), and bicontinuous/multiphasic emulsion. Several studies have reported the increased use of O/W nanoemulsions as a delivery system for encapsulating lipophilic components in pharmaceuticals, highlighting the immense potential of nanoemulsions in contributing to novel TDDS-based advances in pharmaceutical applications [58, 68].

## Methods for characterizing TDDS

The evaluation of delivery efficiency and effectiveness is a very important process in TDDS. There are various methods used for this, depending on the type and purpose of the drug to be delivered. However, the three most common methods involve the use of diffusion cells, tape stripping, and microscopic and spectroscopic examination [81, 82], in which each method makes use of a distinct analysis method. As the drug applied to the surface is absorbed, all these characterization methods are based on the principle of measuring the amount of the drug in each surface layer or storing an imaging material instead the drug to visually confirm the absorption behavior.

### Diffusion cell method

**Fig. 4:**
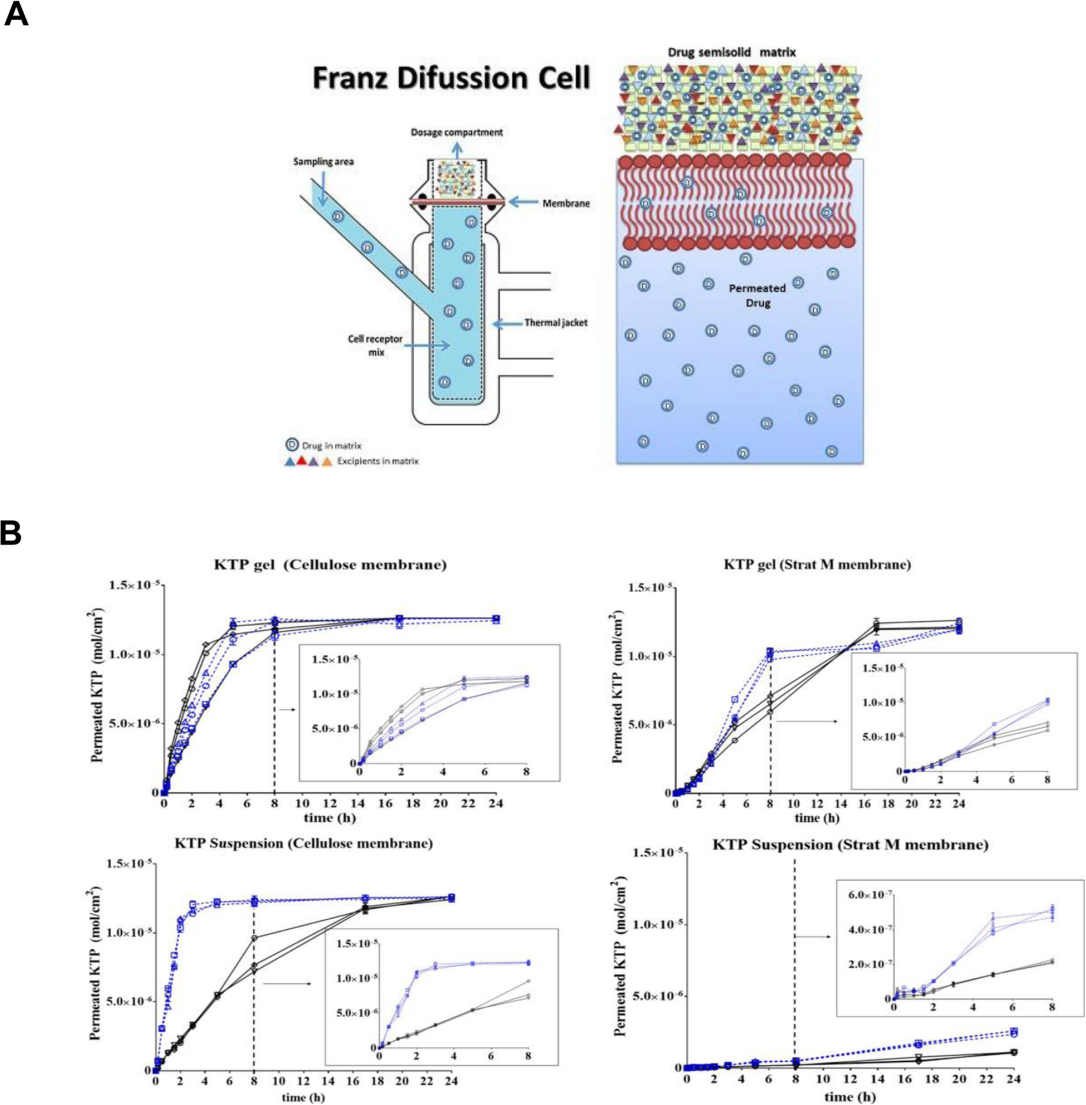
**A** Schematic illustrations of the static Franz diffusion cell. **B** Permeation profiles of ketoprofen (KTP) for 24 h in different conditions of matrix, medium, pH, and type of membrane. **A**, **B** Reproduced from [83], copyright permission by MDPI 2018

Tests employing diffusion cells represent the gold standard in the evaluation of TDDS, with Franz diffusion cells being the most common used setup (Fig. 4) [84, 85]. This technique determines important relationships among the skin, active pharmaceutical ingredients, and the nature of the formulation. The diffusion cell consists of a chamber for drug application, a membrane within which the drug may diffuse, and an acceptor media chamber from which samples may be investigated. Diffusion cells are categorized into two main classes, namely, static and flow-through cells. In static cells, as in the popular Franz diffusion cell, the donor, the membrane, and the acceptor modules could be placed either vertically or horizontally. There are Franz cells that open from above; therefore, the measurement runs under conditions of atmospheric pressure. However, most of these cells are closed from the top, leading to increased pressure, which translates to an overestimation of penetration values. Nowadays, “hand-sampler” Franz diffusion cells have been replaced by systems connected to an automated sampler. These automated sampling systems facilitate the work of researchers and reduce errors from manually conducted experiments.

### Tape stripping

**Fig. 5:**
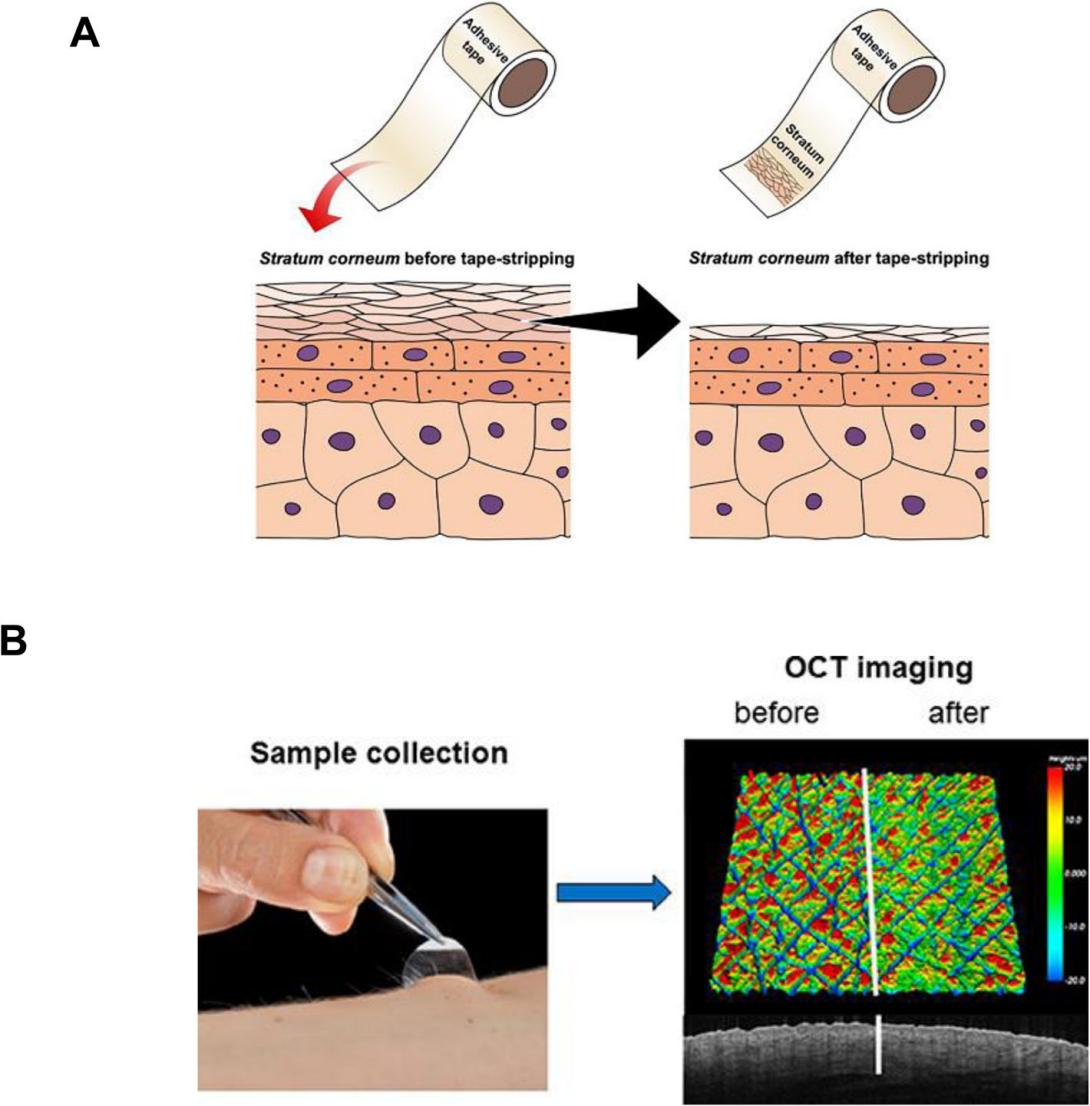
**A** Diagram illustrating the process of skin tape stripping. **B** Imaging of a 4-mm volar skin surface area of a healthy forearm with optical coherence tomography (OCT). **A** Reproduced from [66], copyright permission by Springer Nature 2021. **B** Reproduced from [74], copyright permission by Frontiers Media 2019

Tape stripping is a commonly used minimally invasive method to test the penetration of topically applied formulations through the SC, where a layer of the SC is removed with an adhesive tape followed by examination of the skin layer on the adhesive tape (Fig. 5) [74, 83, 86, 87]. The tape stripping process is performed after an appropriate incubation time post topical application of the test composition. The composition may be removed or left on the skin to provide the original amount of components to be used during the measurement. The adhesive tape is placed on the skin surface and is always removed from the same selection. It is important that the adhesive tape is always flattened with the same force as the roller to eliminate the effect of creases and recesses on tape stripping. In addition, the removal rate is an important factor. The slower the adhesive tape removal rate, the higher the adhesion of the SC to the patch, which increases the amount of skin removed from the patch. The removed adhesive tape contains both the SC layer and the active ingredients of the composition used. Several methods can be used to test samples harvested using adhesive tape. High-performance liquid chromatography (HPLC) analysis produces quantitative results, whereas spectroscopic methods produce semiquantitative insights. During HPLC analysis, the test material on the adhesive tape is extracted and analyzed on chromatographic separation. It is also possible to detect active substances using atomic absorption spectroscopy. However, the most prevalent method used to characterize skin harvested by tape stripping is attenuated total reflectance-Fourier transform infrared spectroscopy (ATR-FTIR). These spectroscopic measurements are based on sample irradiation, and changes in oscillations and bonding angles between atoms due to the absorption or scattering of infrared rays. The change in radiation on passing through the sample is measured by plotting the transmitted radiation as a function of wavelength/wavenumber. This analysis yields a spectrum that could be analyzed for both qualitative and quantitative information. Therefore, the depth of penetration is determined by the wavelength of the infrared radiation, the refractive index of the ATR crystal, and the measured material and angle of reflection. Tape stripping combined with ATR-FTIR spectroscopy is suitable for detecting a variety of exogenous substances in specific layers of the SC. However, the difficulty with this method is that characteristic peaks of the substance to be detected often overlap with peaks specific to the skin.

### Microscopic and spectroscopic methods

**Fig. 6:**
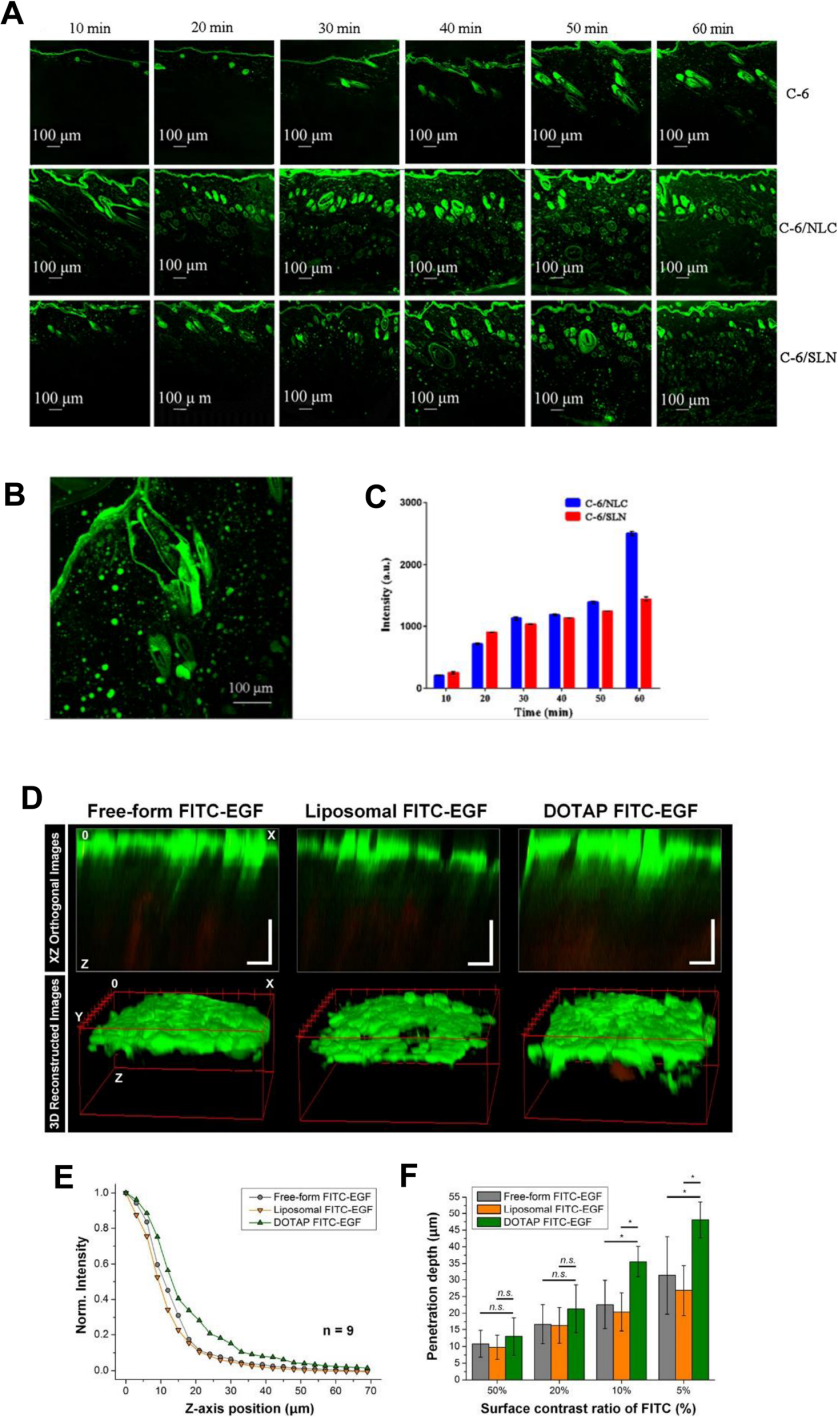
**A** CLSM images (100× magnification) of skin samples treated with free C-6, C-6/NLC, and C-6/SLN. **B** Enlarged CLSM Fig. (200× magnification). **C** Fluorescence intensity in receptor fluid at various times. **D** Reconstructed two-photon images in XZ orthogonal and 3D views. **E** Averaged normalized FITC-EGF signal intensity along the z-axis from the surface to the dermal layer of human skin samples. **F** Penetration depth of FITC-EGF with different thresholds of fluorescence intensity (50, 20, 10, and 5%) measured at the skin surface. **A**, **B**, **C** Reproduced from [88], copyright permission by Springer Nature 2018. **D**, **E**, **F** Reproduced from [89], copyright permission by OSA Publishing 2018

Microscopy-based techniques can also provide important information about the spatial distribution of the drug within different skin layers or shed light on the mechanism of penetration. The two most common modalities of microscopy are confocal laser scanning microscopy (CLSM) and two-photon fluorescence microscopy (2-PFM) (Fig. 6) [58, 68, 71, 72, 74, 80–87, 90].

CLSM is a non-invasive method developed for fluorescence microscopy [88, 91, 92]. In the past few years, CLSM has been widely adopted as a technique to visualize fluorescent model compounds in the skin. CLSM can be used to examine skin structure without destroying tissue samples and is widely employed to evaluate the effect of physical and chemical enhancers on skin permeability. This method can be adapted for use in both in vivo and in vitro conditions. CLSM is used to diagnose common skin dysfunction and identify malignant lesions, along with characterization of keratinization and pigmentation disorders. CLSM can be applied to probe the mechanism underlying the promotion of transdermal transport by nanoparticle formulations. Fluorescent markers (e.g., fluorescein, Nile red, and 5-bromodeoxyuridine) can be included in the encapsulated nanostructured formulations. The therapeutic effectiveness of these formulations can be examined by CLSM to determine the penetration profile of these fluorescent markers across skin tissue or skin appendages.

In addition, 2-PFM has become an important tool for imaging skin cells [89]. This setup commonly uses a Ti-sapphire laser as the excitation source. In single-photon fluorescence, a fluorescent photon is generated when a high-energy photon excites the fluorophore and increases the energy level of one of its electrons to an excited state. In two-photon excitation, the combined energy transfer of two low-energy photons is sufficient to raise the same electron to a high energy level. The setup of a two-photon microscope is very similar to that of a CLSM, with two major differences. The 2-PFM setup works with an adjustable Ti-sapphire high-frequency pulsed laser, which emits red and near-infrared rays in the wavelength range of 650–1100 nm. Another significant difference is that there is no pinhole in front of the detector. The most relevant advantage of 2-PFM is that the total energy delivered to the specimen is much lower than that of other techniques. In addition, the two-photon excitation phenomenon involves fluorescence excitation of the sample in very small focal volumes, thereby reducing the possibility of photobleaching and photodamage. Skin samples can be studied without cryofixation or sectioning. For imaging of UV-absorbing fluorophores, less scattering and less absorption make deep tissue imaging possible using infrared excitation. The limitations of 2-PFM include the fact that this setup requires relatively expensive lasers and complex cooling systems. It also has a lower lateral resolution than other technologies; however, in practice, the resolution difference is not significant.

## Conclusion and future perspectives

The development of TDDS technology is widely recognized as the development of a mass delivery methodology, which makes it the preferred drug injection modality for transdermal delivery across skin types, while preventing first-pass metabolism and other sensitivities associated with various alternative drug administration routes. In various devices and TDDSs, drugs can be delivered through the skin to the systemic circulation. Drugs are generally reliably and safely delivered through TDDS and are safe and stable from biochemical modifications until they reach the target tissue. TDDS is noninvasive, nonallergenic, and has a set duration and dose delivery method, which allows for uniform distribution of drugs at prescribed and controlled rates. Many new and old formulations are in the process of improving the bioavailability of low-absorption drugs via easy routes of administration that allow large doses to be administered over a long period of time. Therefore, the TDDS technology is growing rapidly in the pharmaceutical field and has succeeded in capturing key value in the market for biomedical applications as a formulation system that can improve drug delivery through topical routes. However, despite extensive research over the past few decades, passive methods such as chemical enhancers have had limited success in increasing transdermal transport of small molecules and have only had a relatively poor ability to increase transport of macromolecules under potentially clinically acceptable conditions. Active transport methods using external devices have more extensively increased the transdermal delivery efficiency of drugs and macromolecules. However, the ability of these technologies to effectively deliver drugs is partially balanced by their reliance on electronic control devices that require energy sources, which limits their utility and cost. Methods of piercing micron-sized pores into the skin, such as microneedles can significantly increase the transdermal delivery of drugs, macromolecules, or particles, but more studies are needed to achieve more safety/low skin damage and cost-effectiveness.

In recent years, the scale of TDDS in the domestic and overseas drug delivery system market has increased, as confirmed through increasing research studies, patents, and commercially available products from many companies and research institutes. In addition, microneedles are attracting great attention even among TDDS modalities, which complement the limitations of the existing simple application type and patch type needles and combine the advantages of microneedles to obtain higher treatment efficiency and effects. For this, manufacturing and commercialization methods are being developed, with judicious implementation of latest technologies, such as 3D bioprinting. Advances in these TDDSs could provide the driving force for controlling prevalence of diseases of cardiovascular and central nervous systems, diabetes, neuromuscular diseases, genetic diseases, and infectious and localized infectious diseases, while spearheading advances in vaccination and supporting patient preference for self-administration of drugs for long-term treatment.

## Data Availability

Not applicable.

## Abbreviations

DDS: Drug delivery system
TDDS: Transdermal drug delivery system
SC: Stratum corneum
MW: Molecular weight
HPLC: High-performance liquid chromatography
ATR-FTIR: Attenuated total reflectance Fourier transform infrared spectroscopy
CLSM: Confocal laser scanning microscope
2-PFM: Two-photon scanning fluorescence microscope
PLGA: Poly(lactic-co-glycolic acid)
